# A clinical decision model for failed adrenal vein sampling in primary aldosteronism

**DOI:** 10.3389/fendo.2024.1497787

**Published:** 2025-01-17

**Authors:** Sophie N. M. ter Haar, Sofie J. van Goor, Eleonora P. M. Corssmit, Arian R. van Erkel, Bartholomeus E. P. B. Ballieux, Olaf M. Dekkers, Michiel F. Nijhoff

**Affiliations:** ^1^ Department of Medicine, Division of Endocrinology and Metabolism, Leiden University Medical Center, Leiden, Netherlands; ^2^ Department of Radiology, Leiden University Medical Center, Leiden, Netherlands; ^3^ Department of Clinical Chemistry and Laboratory Medicine, Leiden University Medical Center, Leiden, Netherlands; ^4^ Department of Clinical Epidemiology, Leiden University Medical Centre, Leiden, Netherlands

**Keywords:** primary aldosteronism, adrenal vein sampling, LAV/IVC index, disease subtype, failed right cannulation, adrenalectomy

## Abstract

**Objective:**

Primary aldosteronism (PA) is a common cause of secondary hypertension with unilateral and bilateral subtypes requiring different treatments. Adrenal vein sampling (AVS) is the gold standard for subtype differentiation but can be unsuccessful by challenging right adrenal vein anatomy. This study aimed to develop a clinical decision model using only measurements from the left adrenal vein (LAV) and peripheral blood (IVC) to differentiate between PA subtypes.

**Methods:**

The retrospective cohort study included 54 PA patients who underwent bilaterally successful AVS. The main objective was to determine optimal cut-off values for the LAV/IVC index, using ROC analysis for subtype prediction. The predictive value of this index was assessed with the Area Under the Curve (AUC). The Youden index calculated cut-off values, targeting a specificity >90% for PA subtype differentiation.

**Results:**

The cohort, averaging 48.5 ± 9.5 years in age, comprised 21 women and 33 men, among whom 26 presented with unilateral and 28 with bilateral disease. LAV/IVC values <1.2 indicated unilateral right-sided disease (specificity 91%, sensitivity 96%, AUC 0.98, 95% confidence interval (CI) 0.95-1.0), values 1.2-2.4 suggested bilateral disease (sensitivity 93%, specificity 64%, AUC 0.85, CI 0.73-0.96), whereas values ≥4.4 predicted unilateral left-sided disease (specificity 93%, sensitivity 60%, AUC 0.85, CI 0.73-0.96). Published literature aligns with our results on cut-off values.

**Conclusions:**

Utilizing the LAV/IVC index, over 70% of unsuccessful AVS procedures due to failed right adrenal cannulation could be interpreted with over 90% certainty regarding the PA subtype, preventing unnecessary resampling and aiding in determining the preferred treatment.

## Introduction

Primary aldosteronism (PA) is a common cause of secondary hypertension and is associated with a higher risk of severe cardiovascular and renal complications as compared to essential hypertension (1–3). Autonomous aldosterone secretion can result from a unilateral aldosterone-producing adenoma or bilateral adrenal hyperplasia. Differentiating between these subtypes is crucial as preferential treatment and associated outcomes differ (4, 5). The preferred treatment for unilateral disease is adrenalectomy, aiming to cure PA. Curation is associated with better overall and long-term outcomes, including improved disease control, quality of life and mortality (5–10). Patients with bilateral disease are generally treated with mineralocorticoid receptor antagonists (MRA). MRA treatment is associated with poorer cardiovascular outcomes, substantial side effects, and reduced quality of life as compared to adrenalectomy (1, 4, 11). Therefore, identifying PA patients that will benefit from surgery is essential.

The gold standard for differentiating unilateral from bilateral PA is adrenal vein sampling (AVS) (12–14). During AVS, both adrenal veins are cannulated, and cortisol and aldosterone levels are measured and expressed as an aldosterone/cortisol (A/C) ratio (15). These ratios will be compared to determine the disease subtype. However, AVS can fail due to difficult anatomy (16). Unsuccessful sampling is mostly the result of failed cannulation of the right adrenal vein (RAV). Despite increasing expertise and success rates, recent findings show that failure rates range from 5% to 30% (12, 15, 17, 18). In case of failed sampling, repeat AVS is recommended; otherwise, the etiology remains unknown, preventing optimal treatment.

This study aimed to determine whether the A/C ratio of the left adrenal vein (LAV) compared to the inferior vena cava (IVC) could predict unilateral or bilateral disease in patients with failed RAV sampling. Using two validation cohorts with successful and unsuccessful samplings and subsequent treatment outcomes, along with a comparison of reported cut-off values from the literature, we aim to provide a clinically usable decision-making model to help clinicians predict lateralization in case of unsuccessful right-sided AVS.

## Methods

### Study design and patient population

We conducted a retrospective cohort study of adult patients with PA who underwent AVS between May 2014 and July 2022 at the Leiden University Medical Center (LUMC), a Dutch tertiary referral center for adrenal disease. Patients with confirmed PA and successful bilateral AVS were included.

Patients were excluded if they had other adrenal diseases like Cushing’s disease or if there were major deviations in the pre-protocol work-up, potentially leading to unreliable AVS outcomes. We quantified the total amount of antihypertensive drugs, including MRA, as the daily defined dose (DDD), ATC/DDD WHO Index 2023. All patients underwent computed tomography (CT) or magnetic resonance imaging (MRI) of the adrenal glands prior to sampling. This retrospective study was approved by the scientific board of the LUMC, code W2020.058, and patients were given the opportunity to object to the use of their coded clinical data.

### Primary aldosteronism diagnosis

The diagnostic work-up for PA starts with the aldosterone-to-renin ratio (ARR) (12). The ARR was calculated as the plasma aldosterone concentration (PAC) in pmol/L divided by the plasma renin concentration (PRC) in mU/L. An ARR >100 pmol/mU along with spontaneous hypokalemia confirmed the diagnosis PA (19). Before May 2015, PAC was measured in nmol/L and the plasma renin activity (PRA) in µg/L/hour; in this scenario an ARR >0.85 nmol/µg/hour and hypokalemia confirmed the diagnosis. If patients did not meet these criteria with an ARR >30 pmol/mU, an additional salt loading test (SLT) was performed, during which two liters of 0.9% sodium chloride (NaCl) were administered in a seated position (20). Plasma aldosterone measurements were taken before and immediately after saline infusion. A consistently elevated PAC >179 pmol/L after SLT confirmed PA (21). During both the ARR and SLT, antihypertensive medication known to interfere with renin and/or aldosterone was substituted with non-interfering alternatives (**Appendix S1**) (12). Potassium was supplemented to maintain normal range (3.5-5.0 mmol/L).

### Hormone measurements

Serum aldosterone and renin were determined using chemiluminescence technology (ImmunoDiagnostic Systems GMBH, Germany), while cortisol levels were measured through electro-chemiluminescence immunoassay (Elecsys Cortisol gen2 ECLIA Roche Diagnostic, Germany). The analytical variation was 4.0%-6.3% for aldosterone, 3.4%-4.3% for renin and 2.5%-4.1% for cortisol. Before May 2015, a DiaSorin Plasma Renin activity RIA (Gammacoat Plasma Renin Activity RIA, CA1553, DiaSorin, Italy) was utilized. The ARR was clinically validated for both aldosterone and renin assays, establishing a cut-off of 31 pmol/mU [equivalent to 1.12 (ng/dl)/(µU/ml)], with a sensitivity of 99% and a specificity of 79% (20).

### Adrenal vein sampling

AVS was performed under continuous intravenous stimulation of synthetic adrenocorticotropic hormone (ACTH) Synacthen^®^ at 50 µg/hour. This ACTH infusion enhances the specificity of AVS by minimizing fluctuations in cortisol levels, assuming symmetrical cortisol secretion, as cortisol functions as an internal control (22). Autonomous cortisol secretion was ruled out with a dexamethasone suppression test in case of clinical suspicion for Cushing’s syndrome or in case of incidental detected adenomas. The right common femoral vein was punctured and a 5F sheath was inserted. Selective catheterization of the left and right adrenal vein was performed under fluoroscopic guidance. At least two blood samples were obtained from each adrenal vein, along with two peripheral samples from the sheath with the tip in the inferior vena cava. Aldosterone and cortisol levels were measured in these blood samples, and the A/C ratios of the LAV, RAV and IVC were compared to determine lateralization and/or suppression. The selectivity index (SI), defined as the cortisol ratio between the adrenal veins and the IVC, was used to assess for sampling adequacy. A SI index of 3-fold greater in both adrenal veins indicated successful bilateral sampling (**Supplementary Table S1**) (23, 24).

### Definition of bilateral and unilateral disease

The A/C ratio was determined by calculating the average of the two samples from each site. To distinguish patients with unilateral disease, the study utilized the lateralization index (LI), (A/C ratio of the dominant vein)/(A/C ratio of the non-dominant vein), and contralateral suppression index (CSI), (A/C ratio non-dominant vein)/(A/C ratio IVC) (25). In our center, a LI ≥ 4 was considered indicative of unilateral disease. However, adrenalectomy was offered from LI ≥3 onwards, given the high likelihood of biochemical cure and clinical improvement. Additionally, a CSI <1 was considered consistent with unilateral disease (**Supplementary Table S1**). Patients failing to meet the criteria for both LI and CSI were categorized as having bilateral disease. The LAV/IVC index was defined as the A/C ratio between the left adrenal vein and the inferior vena cava.

### Definition of cure

Post-operative cure was assessed within the first-year post-adrenalectomy. The definition of biochemical cure, according to the Post-Adrenalectomy Surgical Outcomes (PASO) criteria, was used (5). Biochemical cure was defined as the correction of hypokalemia (if present pre-surgery) and normalization of the ARR post-operatively; when ARR was not normalized, the salt loading test was repeated. In addition, we assessed clinical improvement, defined as improved control of hypertension, a reduction in antihypertensive medication use, and symptom resolution.

### Data collection of literature

A search strategy (**Appendix S2**) was developed using different variations of the keywords ‘primary aldosteronism, ‘adrenal vein sampling’ and ‘subtyping’. The PubMed database was explored based on titles; 22 abstracts were screened. Full texts of the eligible studies were evaluated, and a total of 8 studies were included for the literature overview, focusing on unsuccessful sampling of the RAV and using the LAV/IVC index to predict lateralization.

### Statistical analysis

Baseline characteristics were reported as mean ± standard deviation (SD) or median and interquartile range (IQR) if not normally distributed. Categorical variables were expressed as absolute numbers and percentages. Differences between unilateral and bilateral PA patients were tested using the independent T-test and Mann-Whitney U-test. Additionally, the Kruskall Wallis test was used to compare multiple groups for numerical values, and the Chi-squared test for categorical values (26). Differences between pre- and post-adrenalectomy outcomes in the unsuccessfully sampled group were tested using the Wilcoxon signed-rank test and McNemar test for categorical data. Receiver operating characteristics (ROC) analysis was used to calculate cut-off values for both the LAV/IVC index and A/C ratio, aiming to predict the disease subtype. The predictive value of these indices was measured by the area under the curve (AUC). The ratio with the highest AUC, representing the highest predictive value, was selected for further analyses. The Youden index was used to select the optimal cut-off values, ensuring a specificity over 90%, to limit false positive errors and their clinical consequences associated with misclassifying patients (27). Other optimal cut-off values with different desired specificities (>85% and >95%) were identified and presented in the supplementary. A post-hoc power calculation was conducted. Assuming an α of 0.05 and a power of 0.80, calculations showed that a sample size of at least 26 patients in each group would be necessary to detect a difference between a 90% cure rate in the intervention group and a 50% in the reference group (assuming that half of the patients has unilateral disease). A p-value of ≤0.05 was considered statistically significant. All statistical analyses were performed using IBM SPSS Statistics version 29.0.

## Results

### Patient characteristics

Between 2014 and 2022, 92 patients with PA were identified, of whom 82 underwent AVS. Among these, 54 (66%) samplings were bilaterally successful and included in the study. Of these, 28 patients had bilateral disease, while 26 had unilateral disease. The unilateral disease group exhibited a more severe phenotype of PA with a higher incidence of hypokalemia (85% *vs*. 32%, p<0.001) and a trend towards a higher ARR ratio than those in the bilateral group (353 *vs*. 215, p=0.21). Antihypertensive medication use was higher in the unilateral disease group (4.6 *vs*. 3.6). Both groups showed a substantial prevalence of cardiovascular comorbidities (e.g. chronic kidney disease - defined as reduced eGFR or presence of albuminuria - and left ventricular hypertrophy (LVH) - defined as meeting ECG or ultrasonographic criteria for LVH) at baseline (**Table 1A**).

**Table 1A:** Baseline characteristics of patients with bilaterally successful AVS.

	Overall	Bilateral	Unilateral	P-value
	*N=54*	*N=28*	*N=26*	
**Sex, male (%)**	33 (61.1)	15 (53.6)	18 (69.2)	0.24^c^
**Age (mean ± SD)**	48.5 ± 9.5	46.3 ± 9.3	50.9 ± 9.3	0.07^a^
**BMI (mean ± SD)**	29.9 ± 5.9	30.4 ± 5.9	29.4 ± 5.9	0.57^a^
**Number of antihypertensives before treatment (mean ± SD)**	4.1 ± 3.1	3.6 ± 3.0	4.6 ± 3.1	0.23^a^
**Hypokalemia (%)**	31 (57)	9 (32)	22 (85)	<0.001^c^
**ARR** Renin in mU/L (mean ± SD) Renin activity (median [IQR])	284.2 (386.4) 1.49 [1.1-2.9]	215.2 (331.7) 1.39 [1.0-2.4]	353.1 (430.0) 1.59 [no range]	0.21^a^ 0.66^b^
**Aldosterone before SLT (mean ± SD)**	761.6 (385.8)	766.6 (211.5)	753.9 (571.0)	0.93^a^
**Aldosterone after SLT (mean ± SD)**	716.4 (1128.5)	455.2 (171.6)	1106.4 (1705.3)	0.07^a^
**Cardiovascular comorbidities** Reduced kidney function (%) Left ventricle hypertrophy (%) Presence of albuminuria (%)	17 (32) 9 (17) 16 (30)	9 (32) 4 (14) 8 (29)	8 (31) 5 (20) 8 (31)	0.91^c^ 0.62^c^ 0.16^c^
**Adequate controlled hypertension, yes (%)**	10 (19)	6 (21)	4 (15)	0.57^c^

BMI, body mass index; ARR, aldosterone renin ratio; SLT, salt loading test; SD, standard deviation; IQR, interquartile range. ARR measured in pmol/mU or renin activity in nmol/mcg/hour, number of antihypertensives reported in DDD (daily defined doses). Reported as mean ± SD, median [IQR] or N (%). ^a^t-test. ^b^Man Whitney U -test, ^c^chi square.

Adrenal imaging revealed abnormalities in 58% of the unilateral disease group and 32% of the bilateral disease group (p = 0.01). In patients with unilateral disease, imaging showed both ipsilateral and contralateral abnormalities, including adenomas and hyperplastic adrenal glands. In the bilateral disease group, no abnormalities in both adrenal glands were seen, but unilateral abnormalities were observed in 10 patients on either the left or right side. Full details are provided in **Supplementary Table S2**.

Adrenalectomy was performed in 22 patients with unilateral disease, surgery was recommended for an additional 3 patients but not (yet) performed. Post-adrenalectomy, 93% of the unilateral left group and 100% of the unilateral right group achieved biochemical cure of PA (**Table 1B**). Symptom improvement, particularly better cognitive functioning such as improved concentration, was observed in 53% of the patients. Antihypertensive medication use could be reduced in nearly all patients (95%) and fully eliminated in 13% (**Table 1B**). Follow-up data was not available for three patients: one was lost to follow-up, and two patients no longer had hypertension, antihypertensive medication use, or symptoms, but their clinicians did not verify biochemical cure (**Supplementary Figure S1**). Regarding histology, the pathology of the adrenal glands revealed adenoma in 14 patients and hyperplasia was found in 14 patients. The observed pathological variants of aldosterone-producing adenomas in our cohort were KCNJ5 (N=5), ATP1A1 (N=5), and CACNA1D (N=4) mutations.

**Table 1B:** Sampling and treatment outcomes of patients with bilaterally successful AVS.

	Overall	Bilateral	Unilateral	P-value
	*N =54*	*N=28*	*N=26*	
**Outcome AVS (N, %)** Bilateral disease Unilateral left-sided disease Unilateral right-sided disease		28 (52)	15 (28) 11 (20)	
**Outcome adrenal imaging (%)** Normal adrenal glands Bilateral abnormalities Unilateral abnormalities	30 (55.6) 7 (13.0) 17 (31.5)	19 (67.9) 0 (0.0) 9 (32.1)	11 (42.3) 7 (26.9) 8 (30.8)	0.01^a^
**A/C ratio (median [IQR])** Bilateral disease Unilateral left-sided disease Unilateral right-sided disease	3.9 [2.2-10.7]	3.8 [2.8-9.3]	10.6 [5.5-15.3] 0.9 [0.4-2.0]	<0.001^b^
**LAV/IVC index (median [IQR])** Bilateral disease Unilateral left-sided disease Unilateral right-sided disease	2.1 [1.4-3.7]	2.1 [1.7-3.2]	4.8 [3.2-5.9] 0.6 [0.1-1.0]	<0.001^b^
**Underwent adrenalectomy with available follow-up data (cured, %)** Unilateral left-sided disease (N=14) Unilateral right-sided disease (N=5)			13 (92.9%) 5 (100%)	
**Post-operative biochemical outcomes** Hypokalemia (%) ARR (median [IQR])			*N = 19* 0 (0) 6.2 [2.0-16.7]	
**Post-operative clinical outcomes** Improvement of symptoms, yes (%) Total number of antihypertensives (median [IQR]) Reduced number of antihypertensives (median [IQR]) Adequately controlled hypertension, yes (%)			*N = 19* 10 (53) 1.0 [0.0-2.5] 2.8 [1.8-4.5] 12 (63)	

AVS, adrenal vein sampling; A/C, aldosteron/cortisol; LAV, left adrenal vein; IVC, inferior vena cava; IQR, interquartile range; ARR, aldosterone renin ratio. ARR measured in pmol/mU, number of antihypertensives reported in DDD (daily defined doses). Reported as mean ± SD, median [IQR] or N (%). t-test. Man Whitney U-test, ^a^chi-square, ^b^Kruskal Wallis (unilateral left *vs*. bilateral *vs*. unilateral right).

### Subtyping of primary aldosteronism

**Figure 1:**
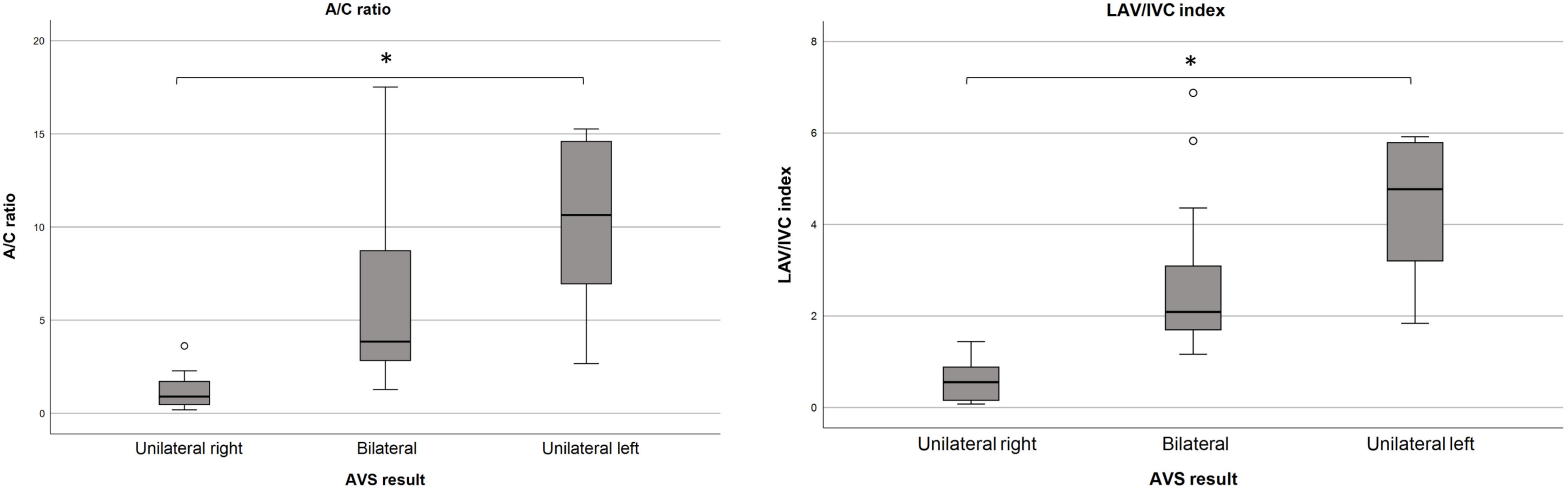
Distribution of the A/C ratio and LAV/IVC index. A/C ratio distribution in the LAV according to disease subtype. Outliers from the unilateral left group (35.3, 41.4 and 47.9) are not displayed. Median A/C ratios (p<0.001); unilateral right 0.9, bilateral 3.8, unilateral left 10.6.B. LAV/IVC index, representing the A/C ratio between LAV and IVC, according to disease subtype. Outliers from the unilateral left group (9.7, 10.8 and 25.1) are not displayed. Median LAV/IVC index (p<0.001); unilateral right 0.6, bilateral 2.1, unilateral left 4.8. A/C, aldosterone/cortisol; LAV, left adrenal vein; IVC, inferior vena cava. * P < 0.001.

The A/C ratio differed between groups (p<0.001): unilateral right-sided (median: 0.9, IQR [0.4-2.0]), bilateral (median: 3.8, IQR [2.8-9.3]) and left-sided disease (median: 10.6, IQR [5.5-15.3]) (**Table 1B**). Similar differences were observed in the LAV/IVC index (p<0.001): unilateral right-sided (median: 0.6, IQR [0.1-1.0]), bilateral (median: 2.1, IQR [1.7-3.2]) and unilateral left-sided disease (median: 4.8, IQR [3.2-5.9]). ROC analysis evaluated the accuracy of the A/C ratio and LAV/IVC index in predicting disease subtype (**Figure 1**). Both ratios showed good predictive ability; however, the A/C ratio had a lower area under the curve. Therefore, this study focused on the LAV/IVC values. For cut-off values off the A/C ratio, see **Appendix S3**.

### Unilateral left-sided disease

**Figure 2:**
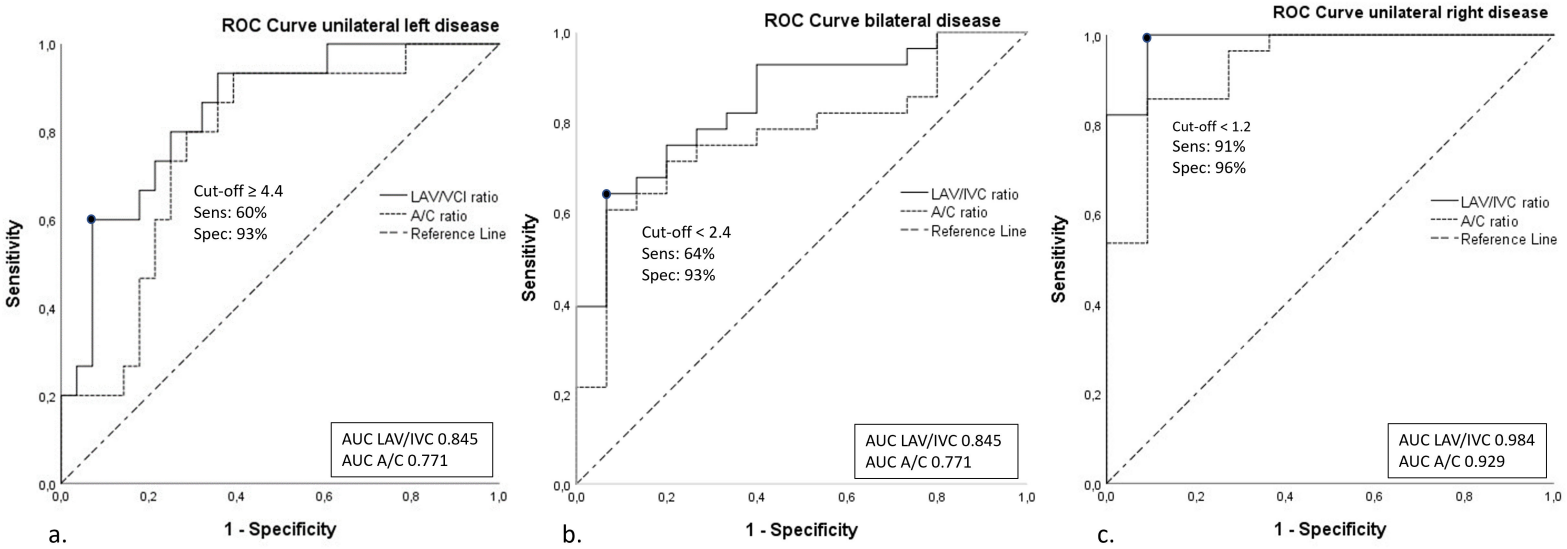
ROC curves of the A/C ratio and LAV/IVC index. ROC-curves of A/C and LAV/IVC cut-off values for **(A)**. Unilateral left-sided disease; **(B)**. Bilateral disease; **(C)**. Unilateral right-sided disease. A/C, aldosterone/cortisol; LAV, left adrenal vein; IVC, inferior vena cava; AUC, area under the curve.

Using the LAV/IVC index, a cut-off value of ≥4.4 predicted unilateral left-sided disease with a sensitivity of 60% and specificity of 93% (AUC 0.85, 95% confidence interval (CI) 0.73–0.96), **Figure 2A**. In other words, when roughly half of the patients have unilateral disease, the LAV/IVC index of ≥4.4 has a positive predictive value of 93% for left-sided disease. However, this cut-off value missed 40% of patients with left-sided disease. Additional cut-off values derived from the same ROC-curve of >4.1 and >5.9 showed a sensitivity of 60% and 27%, with a specificity of 89% and 96%, respectively (**Figure 2A**).

### Unilateral right-sided disease

The optimal cut-off value of the LAV/IVC index for the left adrenal vein was found at <1.2 for predicting right-sided disease with a sensitivity of 96% and specificity of 91% (AUC 0.98, CI 0.95-1.00), **Figure 2C**. Other cut-off values of <1.1 and <1.5 derived from the same ROC-curve, resulted in a sensitivity of 91% and 100% with a specificity of 100% and 82%, respectively.

### Bilateral disease

To predict bilateral disease, a cut-off of value <2.4 was found to have a sensitivity of 64% and a specificity of 93% (AUC 0.85, CI 0.73–0.96). Alternative cut-off values of ≤1.8 and <2.5 derived from the same ROC-curve provided a sensitivity of 39% and 64% with a specificity of 100% and 87%, respectively (**Figure 2B**).

### Clinical decision model

**Figure 3:**
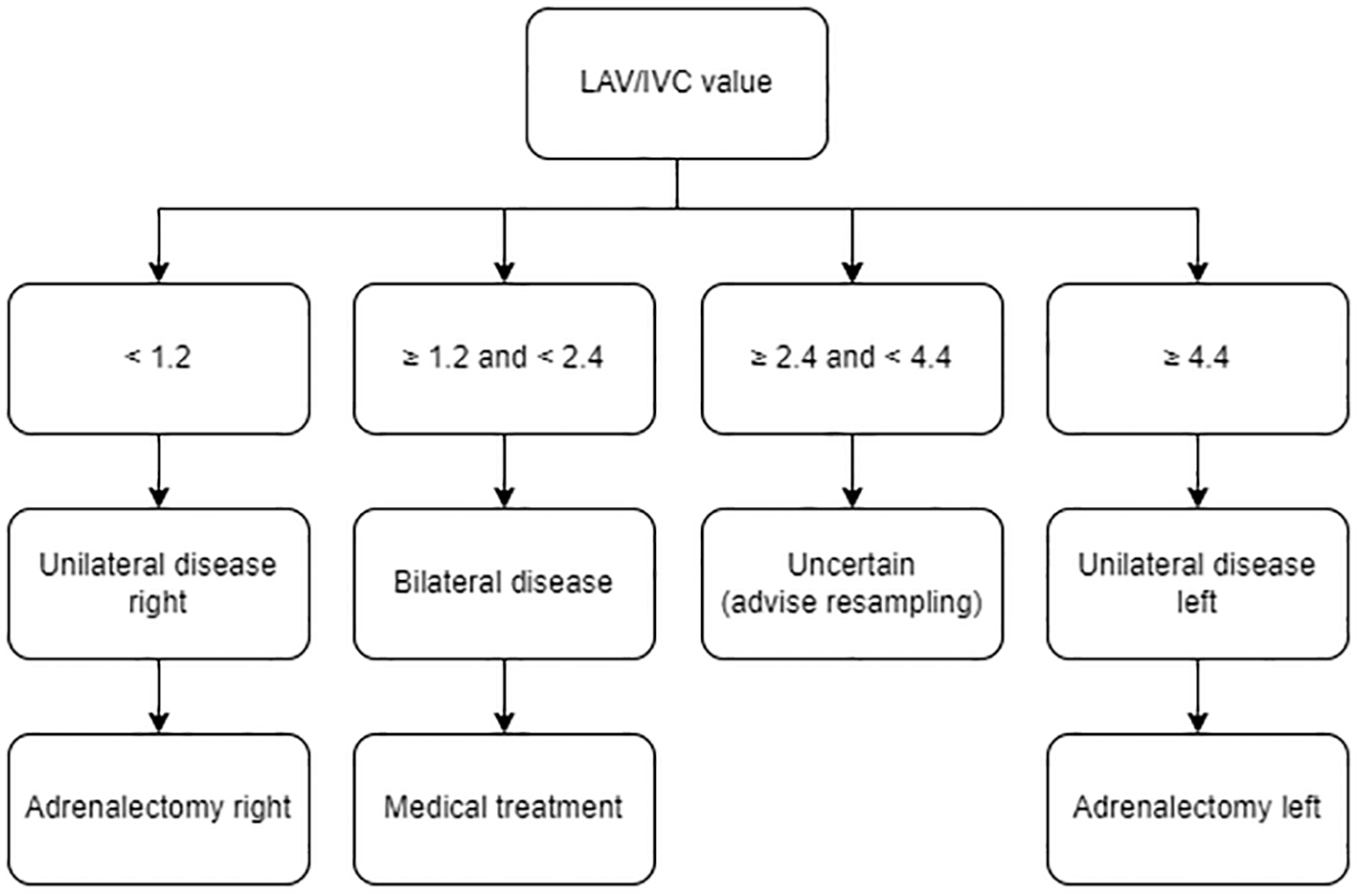
Clinical decision model. Treatment algorithm to interpret AVS sampling data of isolated successful left-sided sampling using the LAV/IVC index, based on specificity >90%. AVS, adrenal vein sampling; LAV, left adrenal vein; IVC, inferior vena cava.

Combining the calculated (optimal) cut-off values, we developed a clinical decision model (**Figure 3**). Additional cut-off values for different specificities (>85% and >95%) for the clinical decision model are provided in **Supplementary Figure S2**. Validating this model on our own study cohort of bilaterally successful sampled patients (N=54), we found that the disease subtype could have been predicted for 74% of the patients. Of whom, 20% were diagnosed with right-sided disease, 35% with bilateral disease, and 19% with left-sided disease. To further validate the tool, we extended the analysis with patients who had unsuccessfully right-sided sampling followed by an adrenalectomy (**Table 2**). During the study period, right-sided sampling failed in 24 patients, of whom 17 were predicted to have unilateral disease by our clinical model. Among those who opted for surgery according to our model’s recommendation, 14/14 (100%) achieved biochemical cure post-adrenalectomy. In this group *(male 79%, age 49 [43-60] years, BMI 30.1 ± 6.1 kg/m^2^)*, differences were observed postoperatively in both the number of antihypertensive medications (in DDD) and the prevalence of uncontrolled hypertension. Additionally, 36% reported a reduction in symptoms, particularly cognitive improvements (**Table 2**).

**Table 2:** Pre- and post-operative data of patients with unsuccessful right-sided sampling, N = 14.

	Pre-operative	Post-operative	P-value
**Sex, male (%)**	11 (79)		
**Age (median [IQR])**	49 [43-60]		
**BMI (mean ± SD)**	30.1 ± 6.1		
**Number of antihypertensives (median [IQR])**	3.3 [1.8-6.5]	0 [0-2.3]	0.03^a^
**Hypokalemia (%)**	12 (86)	0 (0)	<0.001^b^
**ARR** Renin in mU/L (median [IQR]) Renin activity (median [IQR])	136.7 [69.3-494.0] 4.3 [4.3-5.1]	7.1 [0.9-15] 0.5 [no range]	0.05^a^
**Adequately controlled hypertension (N, %)**	2 (14)	12 (86)	0.08^b^
**Presence of symptom, yes (%)** Decrease Increase Similar	5 (36) 8 (57) 1 (7)		

BMI, body mass index; ARR, aldosterone renin ratio; SD, standard deviation; IQR, interquartile range. ARR measured in pmol/mU or renin activity in nmol/mcg/hour, number of antihypertensives reported in DDD (daily defined doses). Reported as mean ± SD, median [IQR] or N (%). ^a^Wilcoxon Signed Rank – test. ^b^Mc Nemar.

### Review of the literature

Various studies have investigated the use of the LAV/IVC index to predict disease subtype. Using the previously described search strategy, 8 articles were identified comparing the LAV/IVC index (28–35). Their cut-off values, including sensitivity, specificity, LI, and SI together with our own data is presented in **Table 3**.

**Table 3:** Comparison of cut-off values with sensitivity/specificity >90%, derived from other literature.

Authors	Number of patients	Cut-off value (LAV/IVC)	Sensitivity %	Specificity %	LI	SI	Stimulated with ACTH
Unilateral left-sided disease							
Wang et al.	222	≥8.6	19%	97%	3:1	3:1	+
**Our study**	**54**	**≥5.9**	**27%**	**96%**	**3:1**	**3:1**	**+**
Kocjan et al.	168	≥5.9	30%	99%	4:1	5:1	+
Strajina et al.	150	≥5.5	45%	82%	4:1	5:1	+
Kocjan et al.	168	>5.5	32%	97%	4:1	5:1	+
Pasternak et al.	36	>5.5	32%	97%	4:1	unknown	+
Lee et al.	121	≥5.5	34%	100%	4:1	5:1	+
Wang et al.	222	≥5.5	49%	89%	3:1	3:1	+
**Our study**	**54**	**>5.5**	**33%**	**93%**	**3:1**	**3:1**	**+**
**Our study**	**54**	≥**4.4**	**60%**	**93%**	**3:1**	**3:1**	**+**
Kocjan et al.	168	≥4.3	51%	95%	4:1	5:1	+
Zibar Tomsic et al.	60	>3.4	5.8%	100%	4:1	5:1	+
Lee et al.	121	≥3.1	74%	82%	4:1	5:1	+
Bilateral disease							
Kocjan et al.	168	<2.5	70%	87%	4:1	5:1	+
Suntornlohanakul et al.	62	<2.4	64%	89%	4:1	5:1	+
**Our study**	**54**	**<2.4**	**64%**	**93%**	**3:1**	**3:1**	**+**
**Our study**	**54**	**≤1.8**	**39%**	**100%**	**3:1**	**3:1**	**+**
Unilateral right-sided disease							
Kocjan et al.	168	<1.25	97%	87%	4:1	5:1	+
**Our study**	**54**	≤**1.2**	**91%**	**93%**	**3:1**	**3:1**	**+**
**Our study**	**54**	**<1.1**	**91%**	**100%**	**3:1**	**3:1**	**+**
Lee et al.	121	<1.0	98%	93%	4:1	5:1	+
Kocjan et al.	168	≤0.5	57%	95%	4:1	5:1	+
Kocjan et al.	168	≤0.5	47%	95%	4:1	5:1	+
Strajina et al.	150	≤0.5	81%	100%	4:1	5:1	+
Lee et al.	121	<0.5	96%	96%	4:1	5:1	+
Wang et al.	222	<0.5	71%	95%	3:1	3:1	+
Pasternak et al.	36	≤0.5	47%	95%	4:1	unknown	+
Zibar Tomsic et al.	60	≤0.37	97.1%	88.4%	4:1	5:1	+
Wang et al.	222	<0.3	71%	97%	3:1	3:1	+
Lin et al.	111	<0.07	40%	100%	2:1	2:1	–
Suntornlohanakul et al.	62	≤0.08	10%	99%	4:1	5:1	+

LAV, left adrenal vein; IVC, inferior vena cava; LI, lateralization index; SI, sensitivity index; ACTH, adrenocorticotropic hormone.

## Discussion

This study provides evidence that left vein AVS data alone effectively classifies PA subtypes in over 70% of cases, reducing the need for resampling or treatment deferral.

Previous studies have shown a nearly 40% discordance between AVS outcomes and anatomical imaging (22). We similarly observed poor concordance in our cohort (**Supplementary Table S2**), which underscores the necessity of AVS for accurately distinguishing unilateral and bilateral PA. Although AVS is a reliable diagnostic procedure, there are important challenges, such as the risk of unsuccessful right-sided sampling, invasiveness, costs, and the associated major medication adjustments, which can lead to a period of uncontrolled hypertension (11–13, 16, 17, 22). Altogether, these factors highlight the importance of strategies to reduce the reliance on resampling.

Our clinical decision model accurately predicts disease subtypes with a specificity of >90% based on left-sided sampling data alone: LAV/IVC <1.2 predicts unilateral right-sided disease, 1.2 to 2.4 predicts bilateral disease, and ≥4.4 predicts unilateral left-sided disease. Resampling is only recommended for LAV/IVC values between 2.4 and 4.4. Optional cut-off values with higher (>95%) or lower (>85%) specificities can be chosen (**Supplementary Figure S2**). Furthermore, our model not only reduces unnecessary invasive resamplings, but also optimizes resource use and lowers healthcare costs. At the LUMC, the second-largest AVS expertise center in the Netherlands (performing 25–50 AVS procedures annually with a success rate of 66%), approximately 30% of the AVS procedures require resampling. Using our model, over 70% of failed procedures can still be interpreted, reducing the resampling rate to 9 per 100 cases, translating to an annual cost saving of approximately €35,000 (36).

Our study is the first to develop a complete clinical model based on cut-off values for predicting lateralization or bilateral disease, incorporating the CSI for right-sided disease. Additionally, it includes a comparison of reported cut-off values from existing literature. In contrast to our study, the clinical tool proposed by Zibar Tomsic et al. proposed lower thresholds (<0.37) for right-sided disease and lower thresholds (0.38-0.68) for bilateral disease (35). Differences stem from their exclusion of the CSI, as it was found to have limited value by Young et al. (37). Recent consensus guidelines however, demonstrated the utility of the CSI for subtyping PA and therefore, it was implemented in our study (12, 15, 18, 38–40). The 100% biochemical cure rate observed in our cohort for right-sided disease - based on the LAV/IVC index - supports the inclusion of the CSI. Furthermore, could their focus on high sensitivity, while we prioritized specificity to avoid misclassification, explain the differences in cut-off values.

While thresholds like LI ≥4 are widely used in the international literature, our model considered LI ≥3 for offering adrenalectomy. This was based on clinical evidence suggesting a high likelihood of cure or significant symptomatic improvement, even at lower thresholds. In our cohort, over 90% of patients with LI ≥3 who underwent adrenalectomy achieved biochemical cure, aligning with published cure rates for LI ≥4. Crucially, shared decision-making plays a critical role in cases with intermediate LI values, as the probability of cure progressively increases with higher LI thresholds.

Importantly, the LAV/IVC index values predicting unilateral or bilateral disease in our study align well with findings from most other studies (**Table 3**). Although previous studies have explored cut-off values for interpreting lateralization using the LAV/IVC index, no consensus has been reached. Pasternak et al. initially proposed cut-off values (>5.5 for unilateral left-sided disease, <0.5 for unilateral right-sided disease) (31). Subsequent studies have tested and adapted these values (28–35). Pasternak’s cut-off value of >5.5 for left-sided disease, showed a sensitivity of 32% and specificity of 97%; our study found comparable values of 33% sensitivity and 93% specificity. Both results are consistent with those found by Kocjan and Wang’s et al. (28, 34). Using the Youden’s index, the optimal cut-off value with a desired specificity >90% was found, yielding ≥4.4, which demonstrated similar sensitivity and specificity as Kocjan’s cut-off value of ≥4.3 (28).

In contrast to many other studies, our cut-off values for bilateral disease were determined at 1.2-2.4 with 93% specificity, while similar studies suggested slightly higher cut-offs with lower specificities (87-89%) (28, 33). For unilateral right-sided disease, our study found a value of <1.2, with a specificity of 91%, aligning with values reported by Lee and Kocjan et al. (28, 29). Discrepancies in reported cut-off values for right-sided disease in other studies may be due to the exclusion of the CSI, potentially missing patients with right-sided disease. Factors such as severe PA in the cohort (Suntornlohanakul et al.) and the use of unstimulated AVS (Lin et al.) could explain their extremely low reported cut-off values. Since our model is developed with ACTH-stimulated AVS, its applicability for centers with unstimulated AVS is questionable. Our results, reflecting similar sensitivities, specificities, and patient demographics as found in existing literature, are representative for the PA population.

Limitations of our study include its retrospective study design and relatively small study group. To address this, we performed an extensive literature analysis that supports our data. Furthermore, subtype determination relied on successful sampling outcomes, whereas other studies used the post-adrenalectomy data to confirm unilateral disease. However, our cure rates >95% post-adrenalectomy, align with published outcomes (5–9), supporting the validity of our findings.

While our high biochemical cure rates highlight the validity of our approach, these reflect biochemical outcomes only, as defined by the PASO criteria. Clinical cure, defined as complete blood pressure normalization without antihypertensive medication, was less applicable to our cohort due to the considerable presence of pre-existing cardiovascular damage (e.g. chronic kidney disease, left ventricular hypertrophy) in our patients at baseline (**Table 1A**). These comorbidities reduced the likelihood of achieving complete clinical cure, even in patients with normalized ARR and correction of potassium levels postoperatively. Instead, we assessed clinical improvement in patients who underwent adrenalectomy, encompassing better-controlled hypertension, reduced medication use, and symptom resolution (**Table 1B**). These findings indicate that adrenalectomy not only resolves biochemical hyperaldosteronism but also leads to meaningful clinical benefits, including enhanced quality of life. This aligns with existing literature, such as Venema et al., who reported significant improvements in both physical and mental health domains after treatment for primary aldosteronism (41).

Our prediction model, validated internally and on unsuccessfully sampled patients, demonstrated its reliability. Notably, 74% of patients with successful bilateral sampling had their disease subtype predicted based on left-sided data alone. Furthermore, the clinical decision model accurately predicted lateralization in 14 out of 14 unsuccessfully sampled patients of whom follow-up data was available, as confirmed by biochemical cure post-adrenalectomy.

Our study presents a reliable prediction model, although there is a 40% chance it may not detect patients with left-sided disease due to the model’s sensitivity of 60%. This raises concerns about potential missed opportunities for beneficial adrenalectomies and, consequently, the cure of PA. However, the model’s specificity exceeding 90% acts as a safeguard, reducing the likelihood of unintended surgeries.

Misclassifying bilateral patients as unilateral often occurs with exceptionally high LAV/IVC values, suggesting significant left adrenal gland overproduction. However, even in cases of misclassification, adrenalectomy in bilateral patients with this high LAV/IVC can still be beneficial by reducing disease severity and improving quality of life, even without complete cure of the disease (25, 42–45). Certain clinical scenarios could alter the reliability of this model. When in doubt, expert consultation, either locally or through a collaboration such as the European Network Reference, is essential. Future research should focus on validating the model in external cohorts, standardized AVS protocols and the reproductivity in cases of unexpectedly failed left-sided sampling.

In conclusion, our study emphasizes the LAV/IVC index’s utility as a valuable predictor for primary aldosteronism subtype, when right-sided sampling fails. Our proposed clinical decision model, integrating the CSI, defines thresholds and could potentially reduce the need for resampling in over 70% of cases with failed right-sided cannulation. Overall, this model facilitates a more efficient and precise approach to subtype classification in patients with PA.

## Data Availability

The raw data supporting the conclusions of this article will be made available by the authors, without undue reservation.
