# Supplementary material for: A clinical decision model for failed adrenal vein sampling in primary aldosteronism
Source: Front Endocrinol (Lausanne). 2025 Jan 17;15:1497787. doi: 10.3389/fendo.2024.1497787 (PMC11782024; doi:10.3389/fendo.2024.1497787)
Supplement: Supplementary file 1 [file DataSheet1.docx]

**Supplemental File for Review**

*Appendix S1. Medication adaptations:*

For the preparation of the renin/aldosterone measurement the following adaptations where made. Diuretics, including MRAs, were discontinued 4-6 weeks before testing and oral contraceptives four weeks before testing. In addition, ACE-inhibitors, angiotensin II receptor blockers, β-blockers (only α-blockers doxazosin was allowed), calcium antagonists (dihydropyridine) and NSAIDs were discontinued 2 weeks before testing.

*Appendix S2. Search strategy:*

(‘’adrenal venous sampling’’[Ti] OR ‘’adrenal sampling’’[Ti] OR ‘’adrenal vein sampling’’[Ti] OR ‘’Hyperaldosteronism’’[Mesh] OR ‘’hyperaldosteronism’’[Ti] OR ‘’primary hyperaldosteronism’’[Ti] OR ‘’primary aldosteronism’’[Ti]) AND (‘’subtyping’’[Ti] OR ‘’subtypes’’[Ti] OR ‘’subtype’’[Ti] OR ‘’bilateral’’[Ti] OR ‘’unilateral’’[Ti] OR ‘’lateralization’’[Ti] OR ‘’lateralized’’[Ti] OR ‘’successful’’[Ti] OR ‘’unsuccessful’’[Ti] OR ‘’failed’’[Ti]).

*Appendix S3. Cut-off values of the A/C ratios*:

A. Cut-off value for unilateral left-sided disease: A/C cut-off >16.0, sensitivity of 80% and specificity of 89%. AUC of 0.77.

B. Cut-off value for bilateral disease: A/C cut-off ≤ 4.0, sensitivity of 61%, specificity of 93%, AUC 0.77.

C. Cut-off value for unilateral right-sided disease: A/C cut-off < 1.6, sensitivity of 73%, specificity of 93%, AUC of 0.93.

*Table S1. Adrenal vein sampling interpretation:*

|  | Definition | Interpretation |
| --- | --- | --- |
| Selectivity index (SI) | PCC_adrenal vein_ / PCC_IVC_ | Value ≥ 3 confirmed correct cannulation of the adrenal vein. |
| Lateralization index (LI) | (PAC_dominant_ / PCC_dominant_) /  (PAC_nondominant_ / PCC_nondominant_) | Value ≥ 3 confirmed lateralized disease of the dominant side. |
| Contralateral suppression index (CSI) | (PAC_nondominant_ / PCC_nondominant_) /  (PAC_IVC_ / PCC_IVC_) | Value < 1 confirmed suppression in nondominant adrenal gland with lateralized disease. |
| LAV/IVC index | PAC_LAV_ / PCC_LAV_) /  (PAC_IVC_ / PCC_IVC_) | Index used to interpret partial AVS data and predict lateralization. |

PCC (plasma cortisol concentration), PAC (plasma aldosterone concentration), LAV (left adrenal vein), IVC (inferior vena cava), AVS (adrenal vein sampling).

*Table S2. Adrenal abnormalities on imaging:*

|  | **Bilateral** | | **Unilateral left** | | **Unilateral right** | |
| --- | --- | --- | --- | --- | --- | --- |
|  | **Normal adrenal glands** | | | | | |
|  | *N=18* | | *N=6* | | *N=5* | |
|  | **Bilateral abnormalities** | | | | | |
|  | *N=0* | | *N=4* | | *N=3* | |
|  | Left adrenal | Right adrenal | Left adrenal | Right adrenal | Left adrenal | Right adrenal |
| **Adenoma** | 0 | 0 | 3 | 1 | 2 | 3 |
| **hyperplastic** | 0 | 0 | 1 | 3 | 1 | 0 |
|  | **Unilateral abnormalities** | | | | | |
|  | *N=10* | | *N=5* | | *N=3* | |
|  | Left adrenal | Right adrenal | Left adrenal | Right adrenal | Left adrenal | Right adrenal |
| **Adenoma** | 3 | 1 | 2 | 2 | 0 | 3 |
| **Enlarged** | 5 | 1 | 1 | 0 | 0 | 0 |

*Figure S1. Study flowchart:*


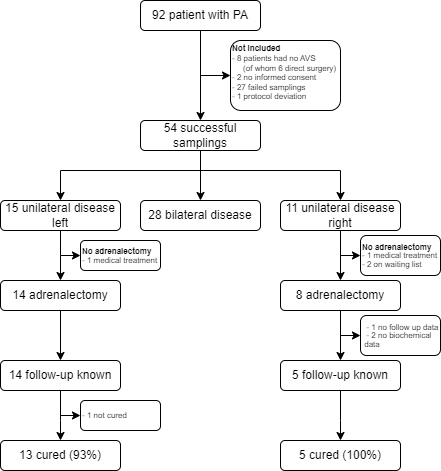


*Figure S2. Clinical decision model with different desired specificities*


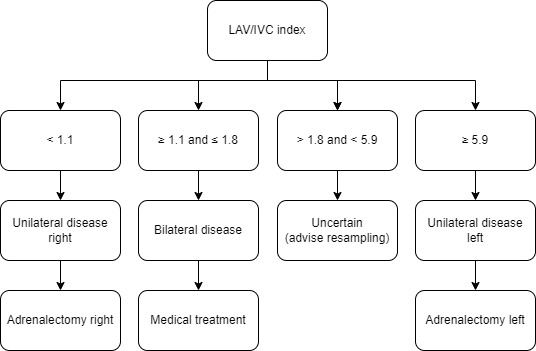


1. Treatment algorithm to interpret AVS sampling data of isolated successful left-sided sampling, using the LAV/IVC index, with a specificity > 95%.


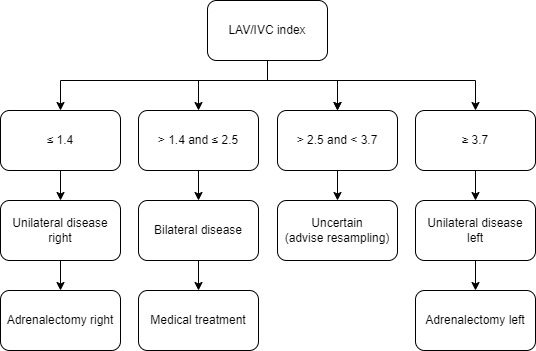


1. Treatment algorithm to interpret AVS sampling data of isolated successful left-sided sampling, using the LAV/IVC index, with a specificity > 85%.
